# Resistance to simian immunodeficiency virus low dose rectal challenge is associated with higher constitutive TRIM5α expression in PBMC

**DOI:** 10.1186/1742-4690-11-39

**Published:** 2014-05-23

**Authors:** Hadega A Aamer, Premeela Rajakumar, Julia Nyaundi, Michael Murphey-Corb

**Affiliations:** 1 Department of Microbiology and Molecular Genetics, University of Pittsburgh School of Medicine, Pittsburgh, PA, USA; 2 Division of Immunology, New England Primate Research Center, Harvard Medical School, Southborough, MA, USA

**Keywords:** APOBEC3G, TRIM5α, Tetherin, SAMHD1, Schlafen 11, Mx1, Mx2, Restriction factors, SIV, Mucosa

## Abstract

**Background:**

At least six host-encoded restriction factors (RFs), APOBEC3G, TRIM5α, tetherin, SAMHD1, schlafen 11, and Mx2 have now been shown to inhibit HIV and/or SIV replication *in vitro*. To determine their role *in vivo* in the resistance of macaques to mucosally-acquired SIV, we quantified both pre-exposure (basal) and post-exposure mRNA levels of these RFs, Mx1, and IFNγ in PBMC, lymph nodes, and duodenum of rhesus macaques undergoing weekly low dose rectal exposures to the primary isolate, SIV/DeltaB670.

**Results:**

Repetitive challenge divided the monkeys into two groups with respect to their susceptibility to infection: highly susceptible (2–3 challenges, 5 monkeys) and poorly susceptible (≥6 challenges, 3 monkeys). Basal RF and Mx1 expression varied among the three tissues examined, with the lowest expression generally detected in duodenal tissues, and the highest observed in PBMC. The one exception was A3G whose basal expression was greatest in lymph nodes. Importantly, significantly higher basal expression of TRIM5α and Mx1 was observed in PBMC of animals more resistant to mucosal infection. Moreover, individual TRIM5α levels were stable throughout a year prior to infection. Post-exposure induction of these genes was also observed after virus appearance in plasma, with elevated levels in PBMC and duodenum transiently occurring 7–10 days post infection. They did not appear to have an effect on control of viremia. Interestingly, minimal to no induction was observed in the resistant animal that became an elite controller.

**Conclusions:**

These results suggest that constitutively expressed TRIM5α appears to play a greater role in restricting mucosal transmission of SIV than that associated with type I interferon induction following virus entry. Surprisingly, this association was not observed with the other RFs. The higher basal expression of TRIM5α observed in PBMC than in duodenal tissues emphasizes the understated role of the second barrier to systemic infection involving the transport of virus from the mucosal compartment to the blood. Together, these observations provide a strong incentive for a more comprehensive examination of the intrinsic, variable control of constitutive expression of these genes in the sexual transmission of HIV.

## Background

Why certain individuals readily acquire sexually-transmitted HIV, while others remain persistently uninfected despite repeated exposure, has confounded scientists since the beginning of the AIDS epidemic. Some studies indicate that differences in host genetics or virus-specific immunity participate in virus control [1-3], but it is unlikely that a single factor is responsible.

It is generally agreed that inhibition of virus replication in the mucosal compartment during the first seven days after sexual exposure is the ideal time to prevent/delay systemic infection [4,5] because the physical barrier imposed by the vaginal and rectal epithelium creates a genetic bottleneck whereby only a few infected cells produce limited amounts of virus [5,6]. We, and others [4], have hypothesized that a second, less studied, barrier also exists that involves the transport of virus from the mucosal compartment into the circulation via the infected cell.

The host innate immune system responds to early replication of the founder population by upregulating type I interferons that, in turn, induce a myriad of interferon-stimulated genes (ISGs) to create an antiviral environment in nearby cells [7,8]. Among the ISGs that have garnered significant attention because they directly inhibit HIV and/or SIV replication are a group of cellular proteins called restriction factors (RFs). To date, six RFs with activity against HIV and/or SIV have been identified (TRIM5α, APOBEC3G (A3G), tetherin, SAMHD1, schlafen 11, and Mx2). Schlafen 11 (SCHL11) and Mx2 are the most recent additions to the RF repertoire. More information is necessary regarding SCHL11’s anti-HIV mechanism [9] and its activity against SIV is as yet unknown. Mx2 inhibits a wide range of primate immunodeficiency viruses [10,11]. In general, RFs are species-specific and less effective against native viral strains [12]. As a result, rhesus macaque TRIM5α potently blocks HIV-1, thereby providing an explanation for the current inability to create an HIV-1: rhesus macaque model [13]. These RFs are not only induced by type I interferons during early infection, they are also constitutively expressed in the naive host [7,9,12,14-16]. Together, these RFs contribute to both the intrinsic and innate defense against lentiviral infection.

Each RF has a different inhibitory activity within the viral life cycle: A3G, TRIM5α, SAMHD1, and Mx2 pose a post-entry block on the first stages of reverse transcription and cDNA import, while SCHL11 and tetherin inhibit viral translation and virion budding, respectively [9,11-13,17-19]. Although currently debated, Mx2 appears to interfere with nuclear uptake of the virus replication complex and/or integration of viral cDNA [11]. However, both events appear to involve the viral capsid.

The importance of these RFs is evident by the co-evolution of viral antagonists (vif, vpu/nef, vpx) that respectively counteract A3G, tetherin, and SAMHD1. The two exceptions are TRIM5α and Mx2. Evasion of TRIM5α by SIV is accomplished by selecting mutations in the N-terminus of the viral capsid containing the macaque TRIM5α binding site [20-23], with specific mutations at this site resulting in changes in binding affinity [23,24]. While it is not currently known whether a viral antagonist exists for Mx2, HIV-1 responds to Mx2 restriction by selecting for mutations in the capsid region (A88) [25]. Interestingly, this activity is more potent in nondividing cells [25].

Most of what is known about the activity of these RFs on virus replication has been discovered by infection studies *in vitro*[9,10,13,19,26,27]. However, an association between expression of several RFs and viral infection/disease *in vivo* has also been observed. In one study, HIV-exposed seronegative individuals had significantly higher A3G levels in PBMC (peripheral blood mononuclear cells) and cervical tissue when compared to healthy controls [28]. A3G also may affect HIV-induced disease progression because in another study, the PBMC of long-term nonprogressors had significantly higher A3G levels than those of non-controllers [29,30]. Higher pre-infection A3G levels were also found to inversely correlate with viral set point [31]. These findings are further supported in rhesus macaques where higher A3G expression was shown to correlate with lower virus loads, increased survival, and protection from subsequent mucosal challenge [32,33].

The evidence for the role of tetherin in controlling virus burden in HIV + humans is conflicting. Whereas higher expression of tetherin was coupled to more rapid disease progression in one study [34]; in another, induction of tetherin by IFNα treatment was associated with a reduction in virus burden [35]. In macaques, transient induction of tetherin was also observed that coincided with viremia and induction of IFNα [36].

The association between TRIM5α expression and virus infection has been best studied in SIV + macaques. Genetic polymorphisms in the TRIM5α B30.2/SPRY domain responsible for binding to the viral capsid have been shown to correlate with susceptibility to both infection and disease with some isolates of SIV but not others [24,37-40]. The direct impact of Schlafen 11, SAMHD1, and Mx2 in controlling virus infection/disease *in vivo* in either system has not yet been shown.

Although these studies support an important role for RFs in the response to SIV/HIV infection and/or disease, it is not clear whether the intrinsic and innate immune response function independently or in a cooperative fashion, and whether one or all of the RFs cooperate to block virus transmission. A better understanding of how the expression of these RFs affects the susceptibility to mucosal exposure will be necessary to fully harness the functions of these RFs for the development of effective vaccines and therapeutics.

To address this issue, we subjected a cohort of eight Indian origin rhesus macaques to weekly low dose rectal challenges with the primary pathogenic isolate, SIV/DeltaB670. Messenger RNA encoding the six RFs known to affect SIV and/or HIV replication (TRIM5α, A3G, tetherin, SAMHD1, SCHL11, and Mx2), together with the ISG Mx1 and IFNγ, was quantified in each animal prior to and at 3-day intervals during the course of the challenges. Although Mx1 has never been shown to be directly associated with inhibition of either HIV-1 or SIV, it is a well-known marker of the type I IFN response and therefore included in our assay [32,41].

Animals were divided into two groups (highly susceptible and poorly susceptible) based on the number of challenges required to induce a systemic infection. Both basal (constitutive) and type I interferon-induced expression of these genes were compared in PBMC, inguinal lymph nodes (ILN), and duodenum to determine the relative association of tissue-specific RF expression to resistance to mucosal challenge. This study provides provocative insight into the function of these RFs in counteracting early virus infection in the mucosa and is the first report of the SCHL11 and Mx2 response to primary SIV infection and their relationship to the other ISGs *in vivo*.

## Results

### Outcome of repetitive low-dose rectal exposures

Eight Indian-origin rhesus macaques received up to 6 rectal challenges at weekly intervals with 250-2500 TCID_50_ of a primary stock of SIV/DeltaB670 until they became viremic. The original source of this isolate was a Nigerian-born sooty mangabey monkey (*Cercocebus atys*) with naturally acquired leprosy that initiated the discovery of SIV (see Additional file 1 for detailed viral lineage) [42,43]. For further monkey studies, SIV/DeltaB670 was amplified *in vitro* in rhesus PBMC (rhPBMC) with a TRIM5^CypA/CypA^ haplotype. This haplotype, although fairly rare in a homozygous state in rhesus macaques, fails to restrict SIV/DeltaB670 replication, with virus growing to high titers *in vitro.* Animals with this haplotype are aggressively infected *in vivo* with peak virion RNA reaching 1×10^9^/ml plasma during the acute viremic episode followed by little reduction in titer (data not shown). This stock is comprised of a complex genetic quasispecies and is highly pathogenic in three species of macaques [44-46].

**Figure 1:**
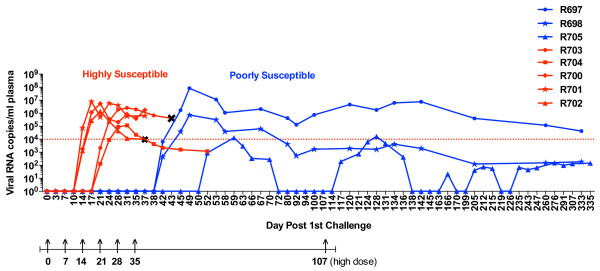
**Outcome of repeated, low-dose rectal challenges with SIV/DeltaB670.** Indian-origin rhesus macaques received weekly low dose rectal challenges. Animals were challenged with 1cc of culture supernatant containing 250 TCID_50_ SIV/DeltaB670 for the first challenge and 2500 TCID_50_ for the remaining 5 challenges. Monkey 705 received a 7th challenge with 2.5 ×10^5^ TCID_50_ 72 days after the 6th challenge. Blood was collected twice a week and copies of viral RNA were quantified in plasma by qRT-PCR using external standards. Animals were divided into two groups based on the number of challenges required for systemic infection: highly susceptible (red); 2–3 challenges (R700, R701, R702, R703, R704) and poorly susceptible (blue); ≥6 challenges (R697, R698, R705). Red dotted line at 10^4^ viral RNA copies indicates the threshold virus load (VL) associated with clinical disease in rhesus macaques infected with SIV/DeltaB670. Arrows indicate time points of challenge. **Χ** = sacrifice for tissue collection.

Longitudinal analysis of the plasma virus burden in these animals is shown in Figure 1. Despite the identical stock/dose employed, the susceptibility to infection varied widely among the animals. Five macaques had detectable viremia after 2–3 challenges (R700, R701, R702, R703, R704; highly susceptible), while three macaques required ≥6 challenges before virus was detected in the plasma (R705, R697, R698; poorly susceptible). Once the animals became infected, the viral set point also varied widely among the animals, in a fashion surprisingly independent of each animal’s susceptibility to infection.

Of particular interest is monkey R705. This animal developed a low-level transient viremia that was only detected 17 days after the 6th low dose challenge. This monkey was re-challenged rectally with a 100-fold higher dose 72 days after the sixth challenge. Despite this dose, the challenge was again followed by minimal transient viremia. Recurrent blips in viremia without further rectal exposures were detected thereafter, with a maximum of 100 copies/ml of plasma persistently detected only after 235 days into the study. In this aggressive model for AIDS, this animal is considered an elite controller, as defined by barely detectable, intermittent levels of virus in the plasma without any other signs of infection or disease [47].

Altogether, these 8 animals displayed the full spectrum of infection and disease previously described for SIV [47-49], with 5 highly susceptible and 3 poorly susceptible to infection following an identical dose/route of virus exposure. Among these, and independent from their susceptibility to infection, all animals except one variably progressed to disease. The eighth animal (R705) became an elite controller.

### Tissue-specific basal ISG expression

Following sexual exposure, relatively few virions pass through the barrier imposed by the mucosal epithelium (6,000 times more infectious SIV/DeltaB670 virions are required to infect macaques intrarectally or intravaginally than intravenously [50]). These establish infectious foci in the rectal and vaginal submucosa [5,51] to become the founder population. Following further amplification, virus subsequently disseminates into the bloodstream via the draining lymph nodes [5,52]. At this point, virus has usually integrated into long-lived resting CD4+ T cells and a persistent infection has been established [53]. One goal of this study was to determine the impact of both constitutive and type I interferon-induced expression of RFs on this process. To address this issue, we quantified both basal and induced expression of A3G, TRIM5α, SAMHD1, tetherin, SCHL11, and Mx2 mRNA 4 days prior to and during the repetitive low dose rectal challenges. Expression of Mx1, an ISG known for its potent restriction of several viruses [41] and frequently employed as a surrogate for type I Interferon induction [32,54] was also analyzed. Quantitation of IFNγ mRNA was also included to examine the impact of RF induction on the adaptive immune response [16]. Three compartments were examined: ficoll-paque purified PBMC, ILN, and duodenal tissue obtained by biopsy.

**Figure 2:**
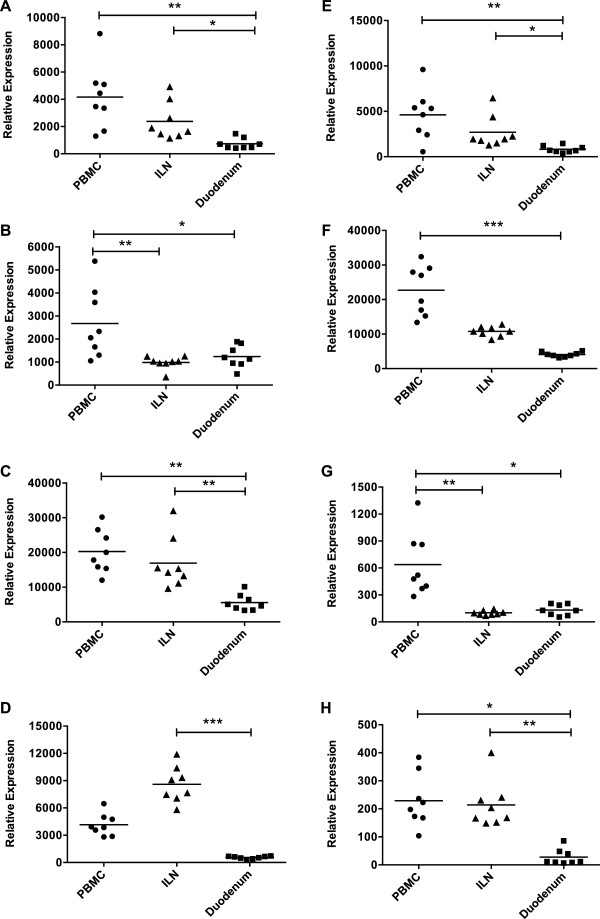
**Basal ISG expression in PBMC, ILN and duodenum.** Relative mRNA levels of **(A)** Mx1, **(B)** TRIM5α, **(C)** Tetherin, **(D)** A3G, **(E)** Mx2, **(F)** SAMHD1, **(G)** SCHL11, and **(H)** IFNγ was determined in PBMC, inguinal lymph node mononuclear cells (ILN), and duodenal tissue biopsies of the eight macaques. All samples were obtained 4 days prior to the first exposure (basal levels) on all animals except monkey R700 from which PBMC samples were obtained on day 0 (day of first exposure). NormFinder [64] was used to determine the most stable endogenous control (TBP, HPRT, β2M, βGus) among the three tissues. All gene expression values were normalized to TBP, the most stable endogenous control. Relative expression was determined using the ΔCt method and the following formula: 1000 x 2^-ΔCt^. Horizontal lines denote mean expression levels in each tissue. Each symbol indicates the mean expression value for one animal. Significance was calculated using the Friedman test with Dunn's multiple comparison test. Asterisks indicate significance at P < 0.05.

As expected, mRNA for all of the ISGs was constitutively expressed in all 3 tissues 4 days prior to virus exposure (Figure 2). Tetherin and SAMHD1 were the most abundantly expressed (Figure 2C,F) and SCHL11 was the least expressed RF in all three tissues (Figure 2G). With the exception of A3G, whose basal expression was higher in ILN than the duodenum (P < 0.01; Figure 2D) basal expression of the other ISGs was higher in PBMC than in the duodenum (P < 0.01). As expected for naive animals, expression of IFNγ at all three sites was less than the other genes (Figure 2H). A heat map of relative ISG expression showed basal expression in PBMC and ILN to be more similar than what was observed in the gut, and further grouped the ISGs according to their similarities in expression (see Additional file 2). Importantly, a strikingly wide range of animal-to-animal variation in the basal expression of these ISGs was observed that was particularly evident in PBMC. Together, these data illustrate that all the ISGs were constitutively expressed in a tissue-specific manner, with higher levels primarily found in PBMC.

### Basal ISG expression and susceptibility to mucosal infection

**Figure 3:**
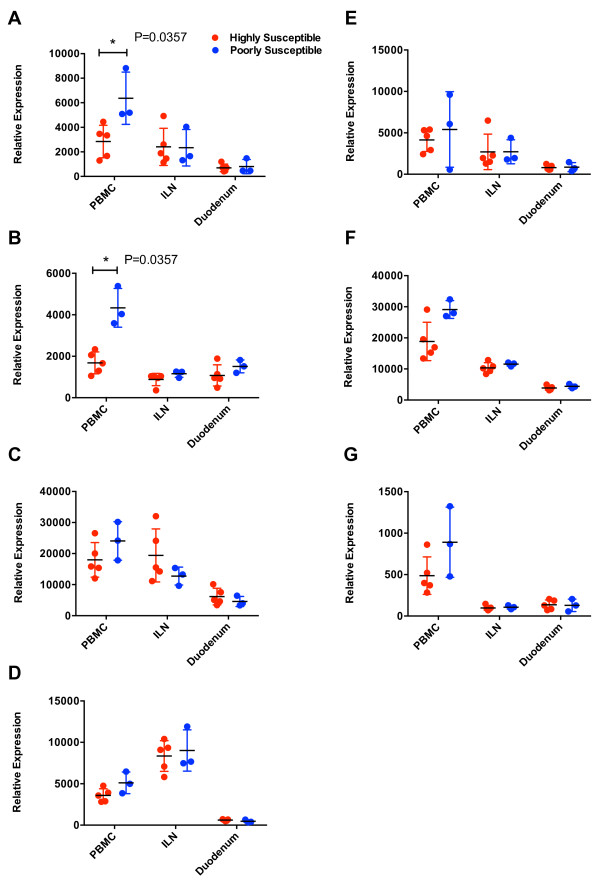
**Statistical analysis of basal ISG expression and susceptibility to infection.** Relative basal mRNA levels of **(A)** Mx1, **(B)** TRIM5α, **(C)** Tetherin, **(D)** A3G, **(E)** Mx2, **(F)** SAMHD1, **(G)** SCHL11 were measured in the PBMC, ILN, and duodenum of the eight macaques, highly susceptible (R700-R704) and poorly susceptible animals (R697, R698, R705). Levels of basal ISG expression for each group were calculated as described in Figure 2. Each symbol indicates the mean expression value for one animal. Significance was calculated by the Mann–Whitney test. Asterisks indicate significance at P < 0.05.

To determine whether there was an association between the basal level of ISG expression and the number of times that an animal had to undergo repetitive challenge to become persistently infected, the average relative expression prior to the first challenge from the two susceptibility groups was statistically compared for each tissue (Figure 3). Significantly higher expression of Mx1 and TRIM5α mRNA was identified in the PBMC of animals poorly susceptible to mucosal infection when compared to that found in the highly susceptible group (Figure 3A,B). These observations could not be explained by differences in the SIV target cells (CD4+ T cells or monocytes) in the PBMC of these animals because, although the absolute numbers varied from animal to animal, the mean numbers in the highly and poorly susceptible groups were not significantly different (data not shown). The mean basal levels of tetherin, A3G, Mx2, SAMHD1, and Schlafen 11 were also higher in the PBMC of the poorly susceptible group but these differences were not significant (Figure 3C-G). In contrast, no significant difference was observed in expression of any of the ISGs between the two groups in either the ILN or duodenum. Even though this analysis was performed on a limited sample size, the magnitude of the observed differences seen for TRIM5α and Mx1 is such that we have power >0.8 to state that these significant differences are genuine, based on determination of Cohen’s *d* as a measure of the Effect Size (ES) seen [55].

**Figure 4:**
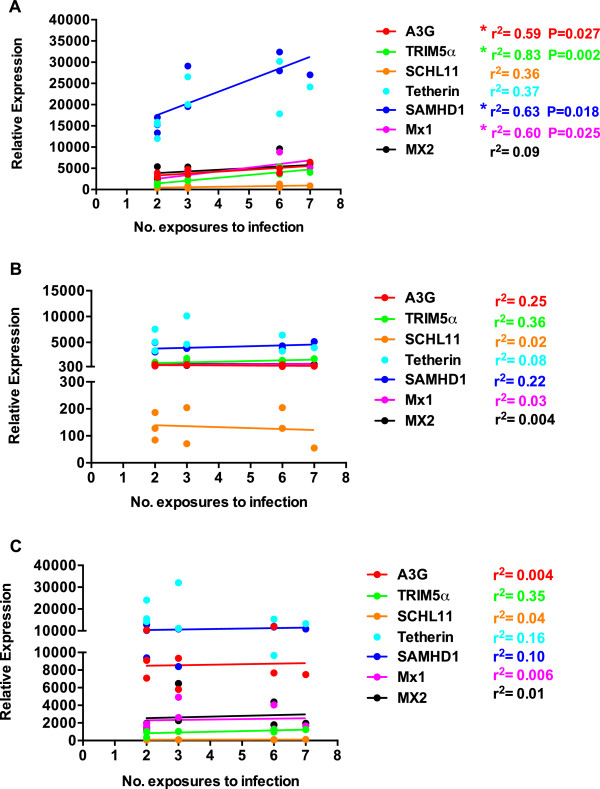
**Linear regression analysis of basal RF expression and resistance to infection.** Basal ISG mRNA levels in **(A)** PBMC, **(B)** Duodenum, **(C)** ILN were calculated as described in Figure 2 and plotted versus the number of exposures required for systemic infection. Each dot indicates mean expression value of one animal. Lines depict linear regression analysis with r^2^ and P values indicated next to each gene. Asterisks indicate significance with P < 0.05.

The relationship between higher basal expression and resistance to mucosal infection was further confirmed by linear regression analysis. Basal ISG expression in each tissue was plotted versus the number of exposures required for systemic infection (Figure 4). This analysis yielded similar findings to that depicted in Figure 3. A significant positive correlation was again observed in PBMC for TRIM5α and Mx1 in addition to SAMHD1 and A3G (Figure 4A). This correlation was especially strong for TRIM5α (r^2^ = 0.83, P = 0.002). Interestingly, despite the strong positive correlation between Mx1 expression and resistance to infection, this relationship was not observed for Mx2. These results are intriguing because, in contrast to Mx2, Mx1 has never been shown to interfere with either HIV-1 or SIV infection. Linear regression analysis further confirmed the lack of an association between basal expression of any of the ISGs in ILN or duodenum and the number of challenges required to induce a systemic infection (Figure 4B,C).

### Basal expression of TRIM5α is stable over time

**Figure 5:**
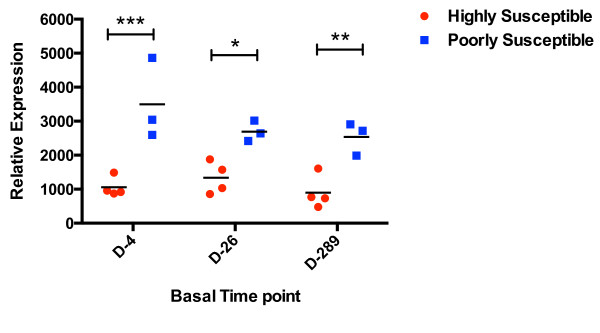
**Basal TRIM5α mRNA levels over time.** Relative basal mRNA levels of TRIM5α was measured 4, 26–33, and 289–298 days prior to the first challenge and plotted for the highly susceptible (R700-R702, R704) and poorly susceptible animals (R697, R698, R705). Values for R703 were omitted due to lack of sample at all 3 time points. GeNorm analysis by the GenEx 6 MultiD software (TATA Biocenter, Sweden) chose TBP and βGus as the most stable genes and all gene expression values were normalized to them using the ΔCt method and the following formula: 1000 × 2^-ΔCt^. Significance was calculated by 2way ANOVA with Sidak’s multiple comparison test. Asterisks indicate significance at P < 0.05. The magnitude of the observed differences seen for TRIM5α is such that we have power >0.8 to state that these significant differences are genuine, based on determination of Cohen’s *d* as a measure of the Effect Size (ES) seen [55].

As an initial attempt to determine whether the varying levels of basal expression of TRIM5α observed among the animals was constant within the individual, we compared the levels of TRIM5α measured 4 days prior to virus exposure to those collected 1 month and 11 months earlier. A comparison of the relative TRIM5α mRNA levels measured 4, 26–33, and 289–298 days prior to the first challenge are shown in Figure 5. At each time point, TRIM5α levels were significantly higher in the poorly susceptible animals compared to those that were highly susceptible. Furthermore, basal TRIM5α levels did not significantly vary over time for each animal (data not shown). These data suggest that basal TRIM5α expression is stable over time at levels that appear to be an intrinsic property of the host.

Mx1 levels were also compared across the 3 time points and unlike TRIM5α, levels varied over time among individual animals and significant differences between the poorly and highly susceptible groups was only seen at the day -4 time point (data not shown).

### Innate immune induction of RFs following virus exposure

We next sought to determine whether variable timing and/or the magnitude of RF induction by virus exposure, both of which could be controlled by intrinsic properties of the host, were associated with the differences in the resistance to mucosal infection. Analysis of ISG and IFNγ expression was measured in PBMC at 3-day intervals during the series of 6 challenges, and in the duodenum 3 days after the first 3 mucosal challenges for each animal. Fold change was determined by dividing relative mRNA levels at each time point with those at baseline (day -4 of the challenge series), with a 2 fold change serving as the minimum threshold for induction.

**Figure 6:**
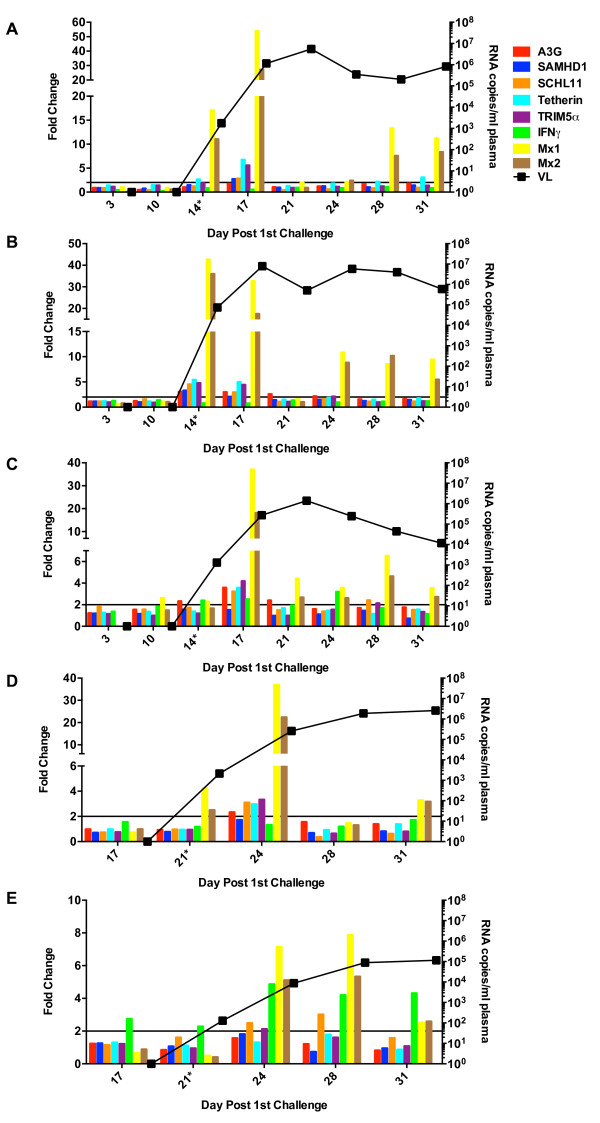
**ISG induction in PBMC of macaques highly susceptible to infection.** The fold change in the relative expression of each RF, Mx1, and IFNγ post-exposure are plotted for susceptible animals **(A)** R700, **(B)** R701**, (C)** R702, **(D)** R703, **(E)** R704**.** Each gene is depicted by a different colored bar as indicated in the figure legend. Plasma virus loads (VL) are shown by the black line. All samples were obtained 4 days prior to the first exposure (basal levels) on all animals except monkey R700 from which PBMC samples were obtained on day 0 (day of first exposure). Fold change in gene induction was calculated by dividing the relative mRNA level at each time point by the basal level obtained prior to first rectal challenge. Values were normalized to the endogenous controls TBP and HPRT. The black horizontal line at a 2-fold change indicates the threshold for the observed induction to be true. Asterisks next to day numbers indicate days of rectal challenge.

**Figure 7:**
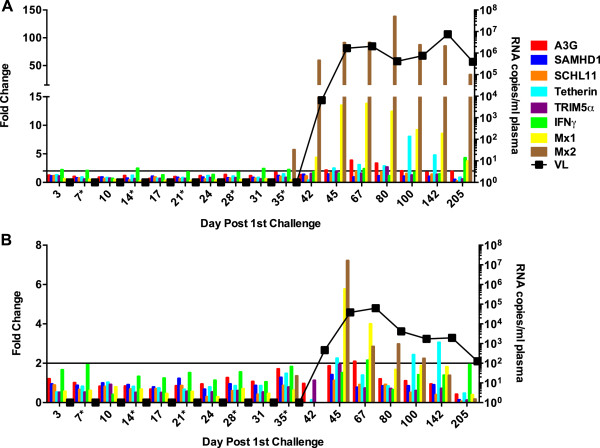
**ISG induction in PBMC of macaques poorly susceptible to infection.** The fold change in the relative expression of each RF, Mx1, and IFNγ are plotted for resistant animals **(A)** R697, **(B)** R698 as described in Figure 6.

**Figure 8:**
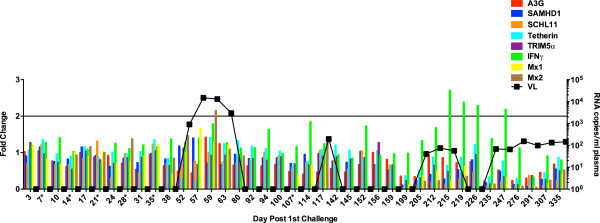
**ISG induction in PBMC of the elite controller.** The fold change in the relative expression of each RF, Mx1, and IFNγ are plotted for the elite controller, R705, as described in Figure 6.

The fold change in ISG and IFNγ expression in PBMC, plotted in relation to plasma virus loads, is shown in Figures 6, 7 and 8. As expected for their role as ISGs, induction of all RFs was observed in most of the animals, with Mx1 and Mx2 expression achieving the highest levels. Induction of these genes was transient, however, and detectable only after virus appearance in the blood. Interestingly, in the highly susceptible monkeys, a transient rebound in Mx1 and Mx2 induction occurred that corresponded with modest increases in plasma virus burden suggesting that several waves of virus production had occurred (Figure 6).

Inexplicably, the pattern of ISG induction varied widely among the 3 animals that required ≥6 challenges to become persistently infected (Figures 7,8). For example, although a transient, modest (<8 fold) induction of Mx1 and Mx2 was observed in monkey R698 in response to virus in the circulation (Figure 7B), a 60–150 fold induction of Mx2 expression was observed in monkey R697 that persisted throughout the maintained high plasma virus burden of 1×10^5^ RNA copies/ml plasma (Figure 7A).

Perhaps the most striking deviation from the pattern observed in persistently infected animals was detected in the elite controller monkey R705 (Figure 8). Expression of the RFs remained unchanged despite the repetitive challenges and transient viremic episodes, with induction levels remaining below the 2-fold threshold. Induction of Mx1 and Mx2 alone was observed at a single time point during the first viremic blip (days 52–63), but this barely achieved the threshold. Induction of IFNγ eventually occurred 205 days into the study that corresponded with the return of virus in the plasma.

### ISG induction in the duodenum

Although analysis of ISG expression in PBMC was surprisingly revealing, we argued that analysis of the response of these genes to incoming virus in the mucosa should tell us more. Although the rectal pouch is the physical site of viral entry, mucosal tissues of the duodenum were biopsied to avoid interference with the weekly rectal challenges. We reasoned that, with the exception that the rectal pouch is more enriched for Peyer’s-like patches than the remaining gut [56], the cellular composition of the gut is similar throughout, with cells in a constant flux of recently activated cells migrating from and mature effector cells returning to the gut via the lymphatics and blood. Thus, both sites (duodenum and rectum) should behave similarly. To assure the validity of these assumptions with respect to ISG induction, we analyzed expression in both duodenal and rectal biopsies 3 days after the 6th challenge of the one monkey that remained uninfected after 6 challenges (monkey R705). A comparison of the values obtained from these tissues showed no significant differences in expression of any of the ISGs in the two mucosal tissues despite the transient appearance of virus in the circulation that followed the 6th challenge (data not shown).

**Figure 9:**
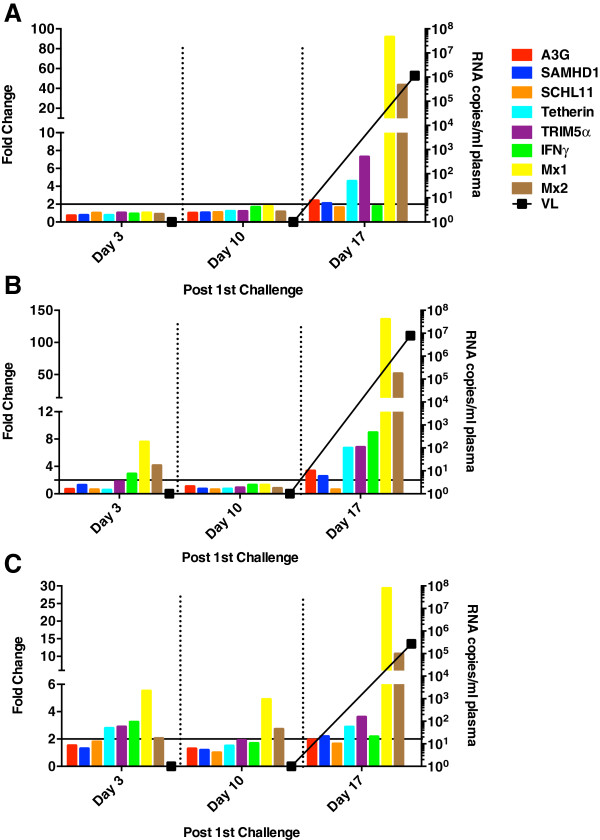
**ISG induction in the duodenum during early challenges.** The fold change in the relative expression of each RF, Mx1, and IFNγ are plotted for the duodenum of animals that became infected during the first 3 challenges **(A)** R700, **(B)** R701, **(C)** R702 as described in Figure 6.

We quantified ISG expression in the duodenum 3 days after the first 3 weekly challenges in all the animals. We discontinued collection of duodenal biopsies after 3 challenges to avoid affecting the health of the animal. A detailed longitudinal examination of ISG induction in the duodenum during the first 3 challenges is shown for the animals that became infected within this timeframe to determine whether ISG induction differed in the gut and blood (Figure 9). The timing and pattern of induction in the duodenum was similar to that observed in PBMC (Figure 6) with two notable exceptions: (1) In two animals (monkeys R701 and R702), a transient, selective induction of Mx1 (5–8 fold) was observed in the duodenum 3 days following the first challenge that was not associated with subsequent viremia, and (2) Induction of Mx1 in the gut of 2 of these animals (monkeys R700 and R701) during viremia was almost 3 times greater than that observed in PBMC at the same time point. ISG induction on day 3 in the duodenum but not PBMC suggests that a transient mucosal infection had occurred in these animals that failed to disseminate to the periphery. The patterns observed in the duodenum for the rest of the cohort over the first 2 challenges showed little to no change in ISG induction, presumably because a transient mucosal infection did not occur in these animals at these time points (data not shown).

## Discussion

The discovery that an array of restriction factors have co-evolved with HIV/SIV to synergistically block replication at various stages of the viral life cycle, and the apparent ability that at least two of these play in blocking cross-species transmission [24,57], supports the argument that a complex system is likely required to limit transmission and control disease within the natural host. The power of these RFs is two-fold: (1) they are constitutively expressed in the immune cells targeted by HIV/SIV, thereby allowing them to function at the moment of infection as intrinsic defense proteins, and (2) they are induced by type I interferons, a function that renders them members of the interferon-stimulated gene family that are essential components of the innate immune system [12,17,22,58]. Together, these functions form synergistic *intrinsic* and *innate* immune weapons of defense against mucosal invasion.

Despite the current knowledge of the function of these RFs in HIV/SIV infection *in vitro*, little is known about the potential role of these genes in controlling virus infection *in vivo*. Moreover, few studies have thoroughly evaluated the role of basal RF expression on the susceptibility to HIV/SIV infection. In this report we describe constitutive (basal) and type I interferon-induced mRNA expression of all 6 known lentiviral RFs (A3G, TRIM5α, tetherin, SAMHD1, Schlafen 11, and Mx2), in addition to another ISG with antiviral properties, Mx1, in a group of macaques undergoing repetitive low dose rectal exposures with the primary isolate, SIV/DeltaB670. The series of weekly low dose rectal challenges resulted in the full range of susceptibility to infection commonly observed for SIV [48,49], with 5 monkeys becoming systemically infected after 2–3 exposures, while 3 monkeys became infected after 6 or 7 exposures. Further, systemic infection resulted in widely variable disease progression patterns that ranged from rapid, immediately progressive disease to elite control. Although the study group contained only 8 monkeys, we reasoned that the balanced diversity in outcome at both levels (transmission and disease) in these animals provided a sound platform with which to examine the contribution of basal and type I interferon-induced RF expression to both.

As expected, all the RFs were constitutively expressed in the animals in PBMC, inguinal lymph nodes, and duodenum prior to exposure. Significant differences in tissue-specific expression were observed, however. Basal levels of all RFs were notably higher in PBMC than in the mucosa, with the exception of A3G, which was more highly expressed in ILN than in PBMC. Strikingly, although maximal relative expression after virus exposure was similar in the PBMC of all eight monkeys, the basal expression of these RFs prior to exposure varied significantly. Statistical analysis of these differences demonstrated that animals more resistant to mucosal infection (≥6 exposures required to establish a systemic infection) had significantly higher basal mRNA levels of TRIM5α and Mx1 in PBMC than highly susceptible animals (2–3 exposures required to establish a systemic infection). Interestingly, this association was observed only in PBMC and not the duodenum or ILN. Moreover, the variable basal levels of TRIM5α, but not Mx1, were held constant with respect to each individual during the year prior to the study, a finding that suggests that control of basal TRIM5α expression is intrinsic to the host. Whether these differences can be genetically explained (e.g., polymorphisms in the TRIM5α promotor region or variation in gene copy number) is currently under study.

Interestingly, our findings are supported by several studies in HIV-exposed humans. Perhaps the most provocative finding was reported in 2009 where a similar analysis of the CAPRISA acute infection cohort demonstrated that PBMC of individuals who remained uninfected had significantly higher basal levels of TRIM5α mRNA at the study start than the PBMC of individuals who subsequently became HIV-1 infected [59]. Higher expression of A3G was also identified in PBMC of exposed uninfected humans than that found in the general population [28,29], but whether this increase was an inherent characteristic of the host or virally-induced was not determined.

Our data provocatively suggest that TRIM5α may potentially affect mucosal transmission of SIV/HIV under the following conditions: (1) Because basal TRIM5α expression in individuals more resistant to infection is higher in target cells at the time of exposure, it may be effective in interfering with the first rounds of early infection (2) However, induction by type I interferons after virus entry appears too little too late; indeed, these events may promote activation and recruitment of target cells to thereby enhance infection [4], and (3) The target cells that provide the critical barrier to infection may not be those that block virus entry and/or allow initial expansion in the mucosal compartment [4], but, rather, those responsible for the dissemination/expansion of virus from the mucosal compartment to the bloodstream. That a marker for susceptibility/resistance to low dose mucosal exposure resides in PBMC rather than in difficult to access mucosal tissues will provide an ideal source for confirming our observations in other macaque and human studies using cryopreserved samples.

It is perhaps noteworthy that because maximum ISG expression levels were essentially the same in all animals, the fold induction calculated as the maximum level over baseline, was associated with animals more susceptible to infection (e.g.; they had lower basal expression). Thus, in the highly susceptible animals, lower basal expression may additionally provide a greater change in the immune environment in response to innate immune induction, thereby allowing greater activation and recruitment of target cells [4]. Conversely, in the elite controller, induction of only Mx2 was detected at a single time point during the first viremic blip. This animal never achieved a virus burden consistent with progressive disease, despite the 100-fold higher dose used as a final challenge. The cause for the failure to respond to virus exposure in this animal is unknown, but is perhaps a critical point for further consideration.

Mx1 induction in the blood was also highest in the highly susceptible animals (7–50 fold), while poorly susceptible (R697, R698) only exhibited a 4–15 fold increase. This finding is consistent with another report of persistently higher Mx1 expression in progressor macaques while little to no Mx1 expression was detected in nonprogressors [54]. Indeed, Mx1 mRNA levels appear to be a reliable indicator of ongoing virus replication, with higher Mx1 levels in chronically infected macaques strongly correlating with increased plasma virus load [54]. The coordinate induction of Mx1 and Mx2 and high virus loads observed in our study is also supported by earlier studies showing persistent IFNα production causing a heightened level of immune activation and T cell apoptosis, thereby facilitating further virus replication and disease progression [16].

Selective induction of Mx1 and Mx2, but not the other RFs, in several of these animals suggests that different cell types or activation pathways may be involved. For example, pDCs in the circulation may have been exposed to free virus particles/proteins either directly by the challenge virus or produced by a limited infection in the mucosal compartment that transiently appeared as free virions in the circulation to selectively activate Mx1 and Mx2. In contrast, induction of the other RFs may require the help of cellular proteins within the infected cell.

Induction of Mx1 in the gut but not the blood of 2 of the highly susceptible animals following the first failed challenge is consistent with a localized infection that was contained within the mucosal compartment. These observations suggest that induction of Mx1 may provide an important signal for the transient presence of virus in the mucosal compartment that fail to establish a productive infection. Whether free virus or the virus-infected cell was subsequently cleared by cells responding to incoming virus or carried to reservoirs to establish a latent infection is unknown at present.

Coordinate induction of RFs was observed in response to systemic infection in animals highly susceptible to infection. This innate response was transient, however, and appeared to be overwhelmed by the tidal wave of virus as it disseminated throughout the body. Similar to other studies [60], induction of the RFs reported here seemed to be a marker of virus replication, rather than an inhibitor. This is further supported in a study examining HIV-1 infected human donor cells where antiviral gene induction did not necessarily correlate with lower virus loads [61].

Important questions are brought to light by our observations that remain unanswered. First, although the stable basal expression of TRIM5α observed in animals suggested that control of constitutive expression is intrinsic to the host, could the basal TRIM5α levels be affected by prior insult to the immune system with an unrelated pathogen? In a recent study in macaques, variable frequencies of α4β7^high^ memory CD4+ T cells were identified in both the blood and gut-associated lymphoid tissue of macaques [62]. Higher populations of these cells correlated with an enhanced *susceptibility* to mucosal infection and higher acute virus load, presumably due to the increase in target cells at the site of mucosal exposure. α4β7 T cell populations fluctuated over time in the tissues, however, with high levels at exposure correlating with susceptibility to infection, whereas those present months earlier did not. Whether this observation relates directly to our own requires further study.

Second, are the same cells responsible for constitutive and type I Interferon-induced expression? If so, what differentially regulates the two? How is this tied in with basal Mx1 (but not Mx2) expression? Are the same target cells critical to mucosal infection found in PBMC also present in the mucosal compartment but in insufficient numbers to have an inhibitory effect? Single cell isolation and detailed analysis of cells infected *in vivo* will be required to address these questions.

Third, at what level do TRIM5 polymorphisms play in our observations? Although differences in the TRIM5α capsid binding site known to control infection with other SIV isolates have no apparent effect in the studies with SIV/DeltaB670 reported here (unpublished) and elsewhere [45], do other polymorphisms, perhaps in the promoter regions that control expression, or variable gene copy number, have an effect? Complete genome sequencing of these animals is underway to address this issue.

While our study has begun to shed light on an important aspect of intrinsic immunity and resistance to infection, there are limitations in our study that are worth highlighting. Although power analysis confirmed our study had an adequate number of animals in the highly and poorly susceptible groups to assure that our observations were valid, additional cohorts of similarly exposed macaques are needed to strengthen our findings. Indeed, although the response to low dose exposure in both vaginal and rectal mucosal lamina propria should be similar, will the vaginal epithelium under strict hormonal control override these events? The data from the CAPRISA study [59] suggest not, but more detailed studies in macaque trials employing vaginal challenge should address this issue. Finally, although we emphasize that the results described in this report are preliminary, they should serve as an important player in guiding future work in the area of the role of lentiviral restriction factors in sexual transmission.

## Conclusions

In summary, analysis of the intrinsic basal and innate immune induced expression of the known lentiviral restriction factors prior to and during low dose repetitive mucosal challenge with SIV in macaques provided the following salient observations to direct future studies:

1. High constitutive (basal) expression of TRIM5α in PBMC, but not gut and lymph nodes, significantly correlated with resistance to mucosal transmission of a primary isolate of SIV.

2. Basal expression of TRIM5α remained constant within the individual, suggesting that control of basal TRIM5α expression is intrinsic to the host.

3. Innate immune induction of RFs in PBMC and duodenum was transient and appeared to be too late to have a major impact on mucosal infection *in vivo.*

4. In contrast to previous studies showing the inhibitory potential on HIV/SIV replication, Mx2 induction had no apparent control on virus levels *in vivo*.

## Methods

### Ethics statement

Eight Indian-origin rhesus macaques, 4 female and 4 male, ranging in age from 3 to 7 years were obtained from an approved vendor (Three Springs Scientific, Perkapsie, PA). The animals were housed and provided environmental enrichment in accordance with “The Use of Nonhuman Primates in Research” and the guidelines of the Association for Assessment and Accreditation of Laboratory Animal Care International (AAALAC) with the approval of the University of Pittsburgh's Institutional Animal Care and Use Committee (Assurance number A3187-01) standards/regulations (Protocol number 1006470). Studies were conducted according to the principals described in The Guide for the Care and Use of Laboratory Animals. Animals were cared for by competent veterinary and animal caretaker staff, fed twice daily and provided daily enrichment. All experimental procedures were performed under ketamine anesthesia and any discomfort or pain was alleviated by appropriate use of analgesic agents at the discretion of the veterinarian. Animals were monitored monthly for signs of disease such as weight loss, respiratory disease and evidence of opportunistic infections. Humane euthanasia was performed in accordance with guidelines as established by the 2007 American Veterinary Medical Association Guidelines on Euthanasia either for tissue collection or once manifestation of clinical AIDS or signs of fatal disease were observed.

### Viral challenge

Each animal was rectally challenged on a weekly basis with 1mL cell free SIV/DeltaB670 (250–2500 TCID_50_). Briefly, sedated animals (10 mg/kg Ketamine i.m.) were positioned in sternal recumbence and inoculated via atraumatic insertion of a 3-ml syringe (lubricated with Surgilube®) approximately 5 cm into the rectum. The inoculum was contained in 1.0 ml of saline. The inoculation was slowly pushed in for a full minute; the syringe left in place for 5 minutes and the animal left in sternal recumbence for a total of 15 minutes. Animals were initially challenged with 250 TCID_50_ of the SIV/DeltaB670 stock but were challenged with 10-fold more virus (2500 TCID_50_) for the remaining 5 challenges. Monkey 705 received a 7th intrarectal challenge with 2.5 × 10^5^ TCID_50_. Challenges were terminated when virus was detectable in the plasma. Blood (3-10 mLs) was collected twice a week from each animal.

### Sample collection

Duodenal biopsies were obtained endoscopically 4 days prior to each of the first three rectal challenges and 3 days later, collected in 1 mL TRIzol, and stored at -80°C. Inguinal lymph nodes (ILN) were surgically removed at these same time points and immediately processed for mononuclear cell purification. 15–18 pinch biopsies of the rectal vault were obtained 4 days prior to the 6th rectal challenge and 3 days later in Monkey R705. The duodenum was specifically sampled due to accessibility by an oral endoscope, ease of sampling without causing harm to the animal, and relevance to understanding mucosal response to SIV infection. The ILN was specifically obtained due to the location and ease of repetitive removal without surgery.

### Plasma virus loads

Viral RNA was extracted from plasma using TRIzol (Ambion) and RT-PCR was performed as previously described using primers specific for the viral long terminal repeat [63]. RT-PCR was performed on a Prism 7700 (Applied Biosystems) using external standards with an 8-log range. Final values were extrapolated from the standard curve and were expressed as RNA copies/ml plasma. Sensitivity threshold for this quantitative assay was ~50 copies.

### MNC purification

PBMC were isolated by Ficoll-Paque (Pharmacia) density barrier centrifugation from rhesus macaque blood samples treated with ACD and were either viably frozen in liquid nitrogen or pelleted and frozen at -80°C. Mononuclear cells (MNC) were isolated from inguinal lymph nodes by teasing and soaking in 10% RPMI 1640 [supplemented with 15% fetal bovine serum, penicillin-streptomycin (100 U/mL), L-glutamine (2 mM), HEPES buffer solution (10 mM)] followed by straining to obtain purified mononuclear cells. After several washes with 10% RPMI, purified MNCs were viably frozen in liquid nitrogen.

### Cellular RNA extraction

Total RNA was isolated from viably frozen PBMC/ILN or frozen PBMC pellets using TRIzol (Ambion). RNA was isolated from the duodenal biopsies by first grinding the tissue with disposable tissue grinders and adding 1mL TRIzol. The Turbo DNA-free kit (Ambion) was used to purify the RNA and remove genomic DNA. RNA concentration was measured on the 2000c Nanodrop spectrophotometer (Thermo Scientific) and RNA purity and integrity was verified using the RNA 6000 Pico kit on the Agilent 2100 Bioanalyzer (Agilent Biotechnologies).

### RF Expression assays

A maximum of 2ug total cellular RNA was reverse transcribed using the High Capacity cDNA Reverse Transcription kit (Applied Biosystems). A total of 25 ng cDNA was used for RT-PCR amplification of each gene and reactions were performed in duplicate. Positive controls and no template controls were included in each assay. Each assay was specific for rhesus macaques and amplified exon-exon regions and did not detect genomic DNA. RT-PCR was performed on the 7900 HT Fast Real-time PCR system (Applied Biosystems) using the recommended reagents (Taqman Fast Universal PCR Mastermix 2×, no AmpErase UNG-Applied Biosystems) and conditions supplied with the Taqman assays. Reaction protocol: 10 uL 2× Mastermix, 1 uL 20× Taqman gene expression assay, 8 uL water, 1 uL 25 ng cDNA. The following are the Applied Biosystems Taqman assay IDs: A3G: Rh02788475_m1, TRIM5α: Rh02788631_m1, Tetherin: Rh02848328_m1, SAMHD1: Rh01122752_m1, Schlafen 11: Rh02885088_m1, Mx1: Rh02842279_m1, Mx2:Rh02842285_m1, IFNγ: Rh02788577_m1, TBP: Rh00427620_m1, HPRT: Rh02827360_m1, β2M: Rh02847367_m1, βGUS: Rh02788764_m1. Since the listed TRIM5α assay detected exons 7–8, which is absent in the TRIMCypA allele in one macaque (R701), we used another TRIM5 probe (Rh02788626_m1) that detected exons 1–2, a non-polymorphic site in both TRIM5α and TRIMCypA. Relative TRIM5 expression levels from both TRIM5 probes showed minor differences and therefore only the TRIM5α probe that detected exon 7–8 was used throughout the study.

All genes were validated for efficiency prior to the start of the assay by creating serial dilutions of a control cDNA sample (see Additional file 3). Linear regression analysis was performed to evaluate assay linearity (R^2^) and efficiency (1–10 ^-1/slope^). All lines had R^2^ values >0.99 indicating linearity. Slopes were between -3.3 and -3.6 and therefore efficiency was >90%. A total of four endogenous controls were used at the start of the assay (TBP, HPRT, β2M, βGus) to determine the most stable genes. Raw RT-PCR data was analyzed first by SDS RQ manager (version 2.3, Applied Biosystems) to obtain the Ct values. DataAssist Software (Applied Biosystems, version 3.01) and NormFinder [64] were used to determine the most stable housekeeping genes and to calculate the 2^-ΔCt^ values. Relative gene expression was determined by the following formula: 1000 × 2^- *Δ*Ct^. Fold change in expression was calculated by dividing the relative expression at each time point by the basal expression (four days prior to the first challenge).

### Statistical analysis

Statistical analysis was performed using Prism 6.0b for Mac OS X and the appropriate statistical tests were listed under each figure. P < 0.05 was considered significant. The statistical power available to us was determined by calculating Cohen’s *d* as a measure of the Effect Size (ES) of our comparisons [55]. The power was then determined using G*Power software [65].

## Abbreviations

SIV: Simian immunodeficiency virus; RF: Restriction factors; ISG: Interferon-stimulated genes; PBMC: Peripheral blood mononuclear cells; ILN: Inguinal lymph nodes; CypA: Cyclophilin A.

## Competing interests

The authors declare that they have no competing interests.

## Authors’ contributions

HAA carried out the experiments and performed statistical analysis of the data. JN carried out animal surgeries/manipulations. HAA, PR, MMC analyzed the data. HAA and PR created the figures. MMC and HAA designed the study and drafted the manuscript. All authors approved the final version of the manuscript.

## Supplementary Material

Additional file 1 **Lineage of the SIV/DeltaB670 challenge stock and relationship to SIVsmE660.** Both SIV/DeltaB670 and SIVsmE660 were derived from sooty mangabey monkey A022 (green box) that was infected by intravenous and subcutaneous inoculation with lepromatous leprosy homogenates from a sooty mangabey monkey with naturally acquired leprosy born in Nigeria (SM A015; white box). Blue boxes indicate monkey-to-monkey passage of SIV/DeltaB670; orange boxes indicate passage of SIVsmE660. Boxes outlined in red indicate a virus isolate. Each arrow indicates virus passage *in vivo* in a monkey or *in vitro* in PBMC. Animal numbers are noted for each passage. The known TRIM5 genotype (CypA/CypA) is noted for RM J943 and RM R245. The TRIM5 genotype is unknown for the other monkeys. SM: sooty mangabey, RM: rhesus macaque, LN: lymph node. Click here for file

Additional file 2 **Heat map analysis of basal tissue-specific RF, Mx1, and IFNγ expression among animals.** Basal gene expression was measured in duodenal biopsies (gut), peripheral blood mononuclear cells (PBMC) and inguinal lymph node mononuclear cells (ILN). All samples were obtained 4 days prior to the first exposure (basal levels) on all animals except monkey R700 from which PBMC samples were obtained on day 0 (day of first exposure). Heat map analysis was performed using GenEx software from MultiD (TATA Biocenter, Sweden). Relative quantity (RQ) values were calculated from ΔCt values using the formula 1000 × 2^-ΔCt^. Heat Maps for gene expression were generated from log_2_ values. RQ values were converted to log_2_ values in GenEx. To classify genes based on expression profiling, log_2_ values were autoscaled for heat map analysis. Click here for file

Additional file 3 **Validation of the linearity and efficiency of Taqman Gene Expression assays.** PBMC cDNA was serially diluted and amplified using pre-developed Taqman Gene Expression assays (Applied Biosystems) and the corresponding Cq (quantification cycle) was plotted at each cDNA concentration. Linear regression analysis was performed to evaluate assay linearity (R^2^) and efficiency (1–10 ^-1/slope^). All lines had R^2^ values >0.99 indicating linearity. Slopes were between -3.3 and -3.6 and therefore assay efficiency was >90%. Click here for file
