# Supplementary figures and images for: Resistance to simian immunodeficiency virus low dose rectal challenge is associated with higher constitutive TRIM5α expression in PBMC
Source: Retrovirology. 2014 May 23;11:39. doi: 10.1186/1742-4690-11-39 (PMC4041354; doi:10.1186/1742-4690-11-39)

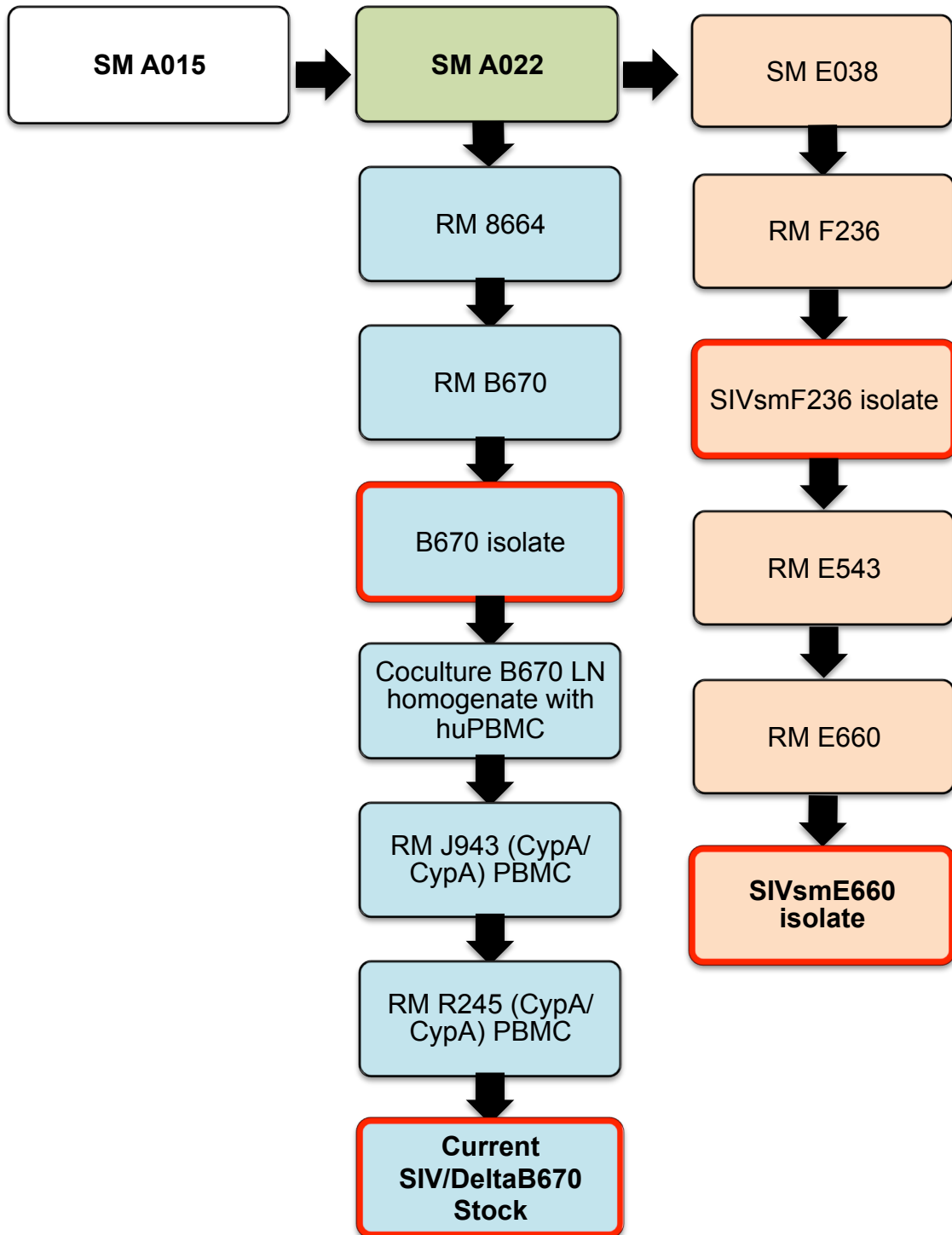

Supplement: Additional file 1 — Lineage of the SIV/DeltaB670 challenge stock and relationship to SIVsmE660. Both SIV/DeltaB670 and SIVsmE660 were derived from sooty mangabey monkey A022 (green box) that was infected by intravenous and subcutaneous inoculation with lepromatous leprosy homogenates from a sooty mangabey monkey with naturally acquired leprosy born in Nigeria (SM A015; white box). Blue boxes indicate monkey-to-monkey passage of SIV/DeltaB670; orange boxes indicate passage of SIVsmE660. Boxes outlined in red indicate a virus isolate. Each arrow indicates virus passage in vivo in a monkey or in vitro in PBMC. Animal numbers are noted for each passage. The known TRIM5 genotype (CypA/CypA) is noted for RM J943 and RM R245. The TRIM5 genotype is unknown for the other monkeys. SM: sooty mangabey, RM: rhesus macaque, LN: lymph node. [file 1742-4690-11-39-S1.pdf]

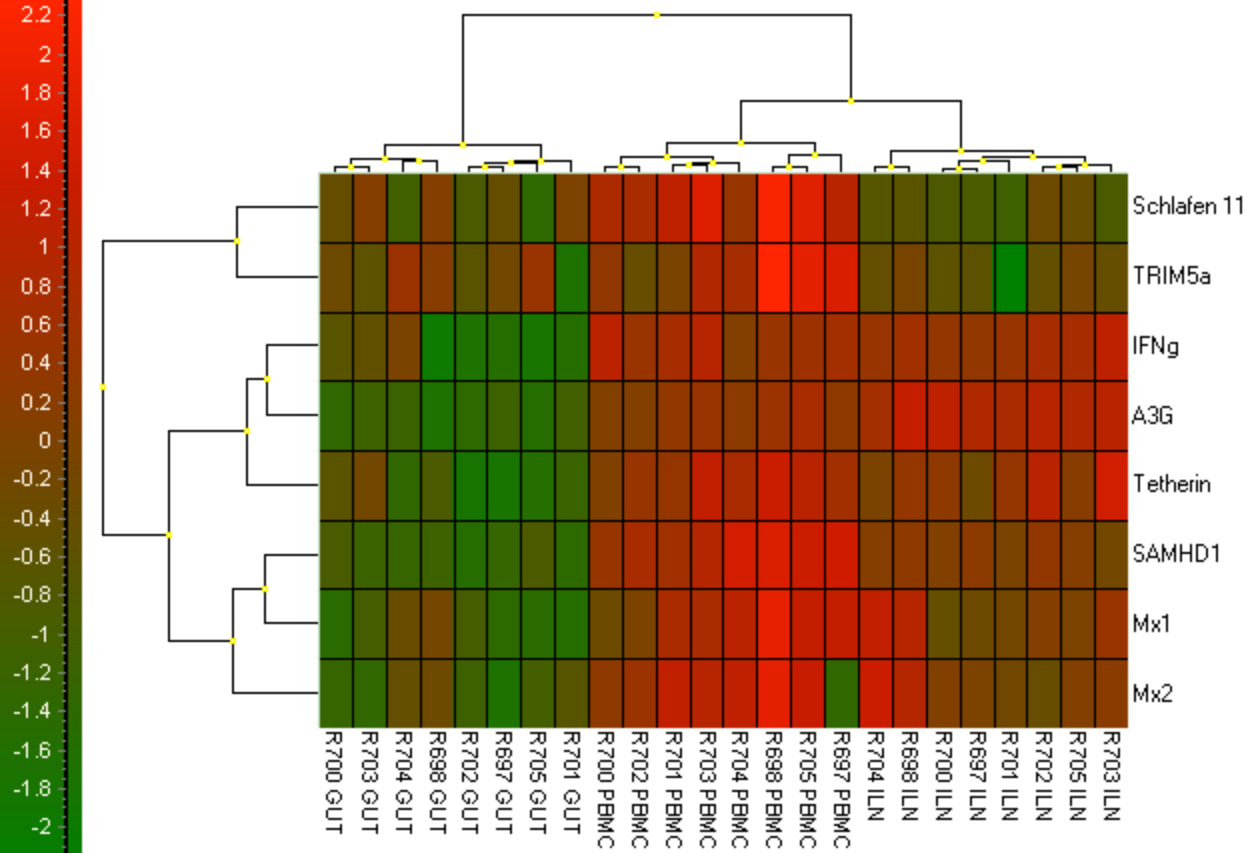

Supplement: Additional file 2 — Heat map analysis of basal tissue-specific RF, Mx1, and IFNγ expression among animals. Basal gene expression was measured in duodenal biopsies (gut), peripheral blood mononuclear cells (PBMC) and inguinal lymph node mononuclear cells (ILN). All samples were obtained 4 days prior to the first exposure (basal levels) on all animals except monkey R700 from which PBMC samples were obtained on day 0 (day of first exposure). Heat map analysis was performed using GenEx software from MultiD (TATA Biocenter, Sweden). Relative quantity (RQ) values were calculated from ΔCt values using the formula 1000 × 2-ΔCt. Heat Maps for gene expression were generated from log2 values. RQ values were converted to log2 values in GenEx. To classify genes based on expression profiling, log2 values were autoscaled for heat map analysis. [file 1742-4690-11-39-S2.pdf]

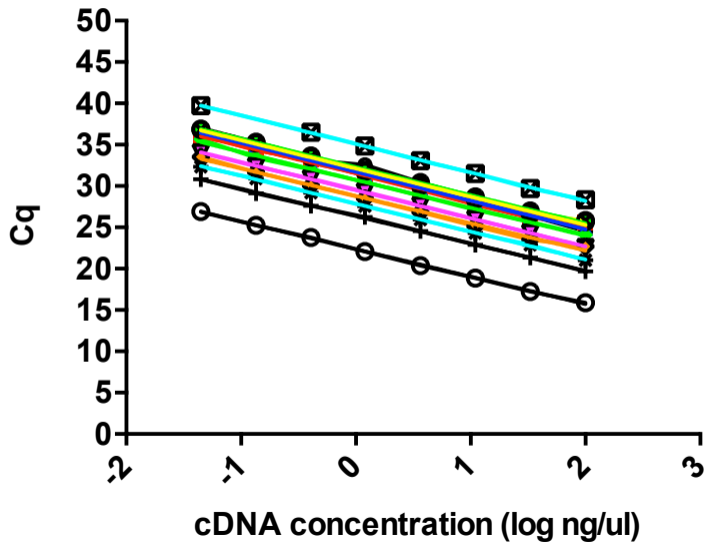

- $\beta$ GUS
- TBP
- HPRT
- $\beta$ 2M
- A3G
- Tetherin
- SAMHD1
- SCHL11
- TRIM5 $\alpha$
- IFN $\gamma$
- Mx1
- MX2

Supplement: Additional file 3 — Validation of the linearity and efficiency of Taqman Gene Expression assays. PBMC cDNA was serially diluted and amplified using pre-developed Taqman Gene Expression assays (Applied Biosystems) and the corresponding Cq (quantification cycle) was plotted at each cDNA concentration. Linear regression analysis was performed to evaluate assay linearity (R2) and efficiency (1–10 -1/slope). All lines had R2 values >0.99 indicating linearity. Slopes were between -3.3 and -3.6 and therefore assay efficiency was >90%. [file 1742-4690-11-39-S3.pdf]
